# Identificación de una nueva mutación en el gen humano xantina causante de la xantinuria tipo I

**DOI:** 10.1515/almed-2021-0059

**Published:** 2021-11-10

**Authors:** Cristina Collazo Abal, Susana Romero Santos, Carmen González Mao, Emilio C. Pazos Lago, Francisco Barros Angueira, Daisy Castiñeiras Ramos

**Affiliations:** Departamento de Análisis Clínicos, Hospital Universitario de Vigo, Vigo, España; Departamento de Medicina Interna, Hospital Universitario de Vigo, Vigo, España; Fundación Pública Gallega de Medicina Genómica, Hospital Universitario de Santiago de Compostela, Santiago de Compostela, España; Laboratory of Metabolic Pathologies, Hospital Universitario de Santiago de Compostela, Santiago de Compostela, España

**Keywords:** hipouricemia, xantinuria, gen de la xantina deshidrogenasa

## Abstract

**Objetivos:**

La xantinuria es una enfermedad rara, de herencia autosómica recesiva caracterizada por la presencia de hipouricemia y elevada excreción de xantina, provocada por el déficit de xantina deshidrogenasa/oxidasa (XDH/XO, EC: 1.17.1.4/1.17.3.2) en el tipo I, o por el déficit de XDH/XO y aldehído oxidasa (AOX, EC: 1.2.3.1) en el tipo II.

**Métodos:**

Describimos una nueva mutación puntual en homocigosis en el gen *XDH* en un paciente con niveles muy bajos de ácido úrico en suero y orina y xantinuria. Aunque el paciente se encontraba asintomático, se objetivaron cálculos renales en las pruebas de imagen.

**Resultados:**

Se detectaron otros casos en su familia, y se le hicieron recomendaciones dietéticas para prevenir futuras complicaciones.

**Conclusiones:**

La xantinuria hereditaria es una patología infradiagnosticada, que se suele descubrir accidentalmente al detectar en un análisis rutinario la presencia de hipouricemia. Es importante que la medicina de laboratorio sepa orientar a los facultativos en su diagnóstico.

## Introducción

La xantinuria (HX) es una patología muy poco frecuente del metabolismo de las purinas causada por un déficit hereditario de la enzima xantina deshidrogenasa/oxidasa (XDH/XO, EC: 1.17.1.4/1.17.3.2), descrita por primera vez en 1954 por Dent y Philpot [1]. Se caracteriza por niveles muy bajos o indetectables de ácido úrico en sangre y orina, con una excreción urinaria normal o baja de urato, y una elevada concentración urinaria de xantina e hipoxantina. Aunque suele ser asintomática, puede detectarse urolitiasis, debido a la elevada excreción de xantina, y miositis causada por la acumulación de xantina. No existe tratamiento, aunque se debe recomendar una dieta baja en purinas e ingesta abundante de líquidos para prevenir complicaciones.

**Figura 1:**
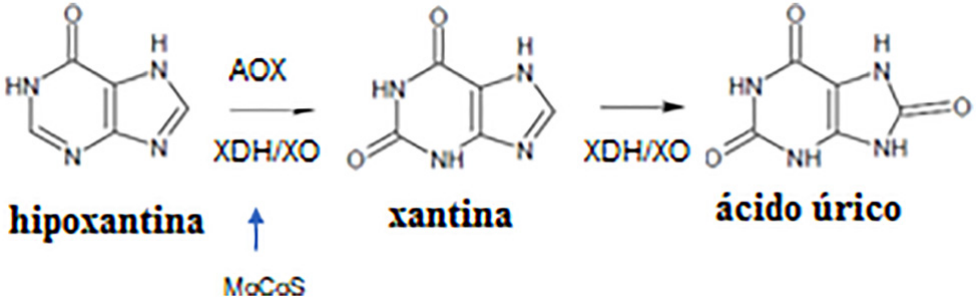
Metabolismo del ácido úrico.

La xantinuria es una enfermedad autosómica recesiva que se divide en tres subtipos. El tipo I (OMIM 278300), causado por mutaciones en el gen *XDH/XO*, en el cromosoma 2p23 [2], [3], [4], [5], [6], se caracteriza por el déficit de XDH/XO, enzima que cataliza la conversión de la hipoxantina en xantina y la degradación de la xantina en ácido úrico, (Figura 1). Por otro lado, mutaciones en el gen del cofactor de molibdeno sulfurasa (*MOCOS*, EC: 2.8), situado en el cromosoma 18q12.2, dan lugar a la xantinuria tipo II (OMIM 603592), caracterizada por el déficit de XDH/XO y AOX (aldehído oxidasa). Ambos tipos, I y II, con un fenotipo asintomático, reciben el nombre de HX clásica [7]. Sin embargo, la xantinuria tipo III se asocia a un fenotipo muy diferente, relacionado con el deterioro neurológico progresivo debido a un fallo en la ruta de la biosíntesis del cofactor de molibdeno, que inactiva la XDH/XO, la AOX y el sulfito oxidasa [8]. La muerte prematura es frecuente, y aquellos que sobreviven presentan convulsiones, tono muscular anómalo, retraso en el desarrollo y luxación del cristalino.

Ichida y col. realizaron una revisión de las mutaciones asociadas a la xantinuria tipo I y II en la última década [3]. Recientemente se han descrito otras mutaciones [5, 9, 10]. Describimos una nueva mutación del gen *XDH/XO*, identificada en un caso de xantinuria tipo I.

## Materiales y métodos

### Paciente

Varón de 65 años con hipouricemia grave detectada en un análisis rutinario, presente al menos en los cuatro últimos años. Altura: 167 cm; peso: 73,5 Kg, IMC: 26,4 kg/m^2^; PA: 138/90 mmHg; FC: 90 ppm. En el examen físico no se encontró ninguna anomalía, en su historial médico figuraba una revascularización por enfermedad cardíaca isquémica crónica y una herniografía inguinal bilateral. No existía consanguinidad entre sus progenitores, ni antecedentes familiares de cálculos renales. Su medicación habitual era bisoprolol, atorvastatina/ezetimiba y pantoprazol. Nunca había tomado fármacos antihiperuricemiantes.

En su analítica, los niveles séricos de ácido úrico eran inferiores al límite de detección de la técnica (<5,95 μmol/L), presentando una función renal normal (aclaramiento de creatinina >90 mL/min/1,73 m^2^). El análisis de orina confirmó una baja excreción de purinas, incluyendo el ácido úrico (53,6 µmol/24 horas).

La ecografía renal mostró unos riñones de tamaño, morfología y ecogenicidad normales. En el riñón derecho se visualizaron dos imágenes hipoecoicas (7 y 9 mm) compatibles con litiasis renales, sin repercusión en el sistema excretor.

**Figura 2:**
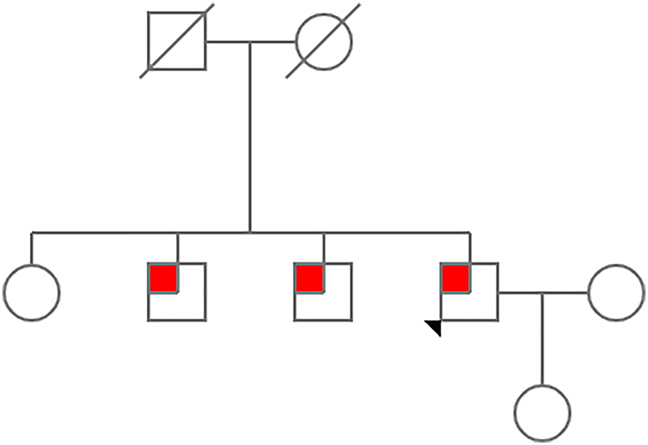
Árbol genealógico.

En sus familiares de primer grado, detectamos niveles bajos de ácido úrico en al menos dos de sus hermanos (Figura 2). Uno de ellos también mostraba niveles de xantina y ácido úrico similares en orina de 24 horas (Tabla 1).

**Tabla 1:** Valores de purina y pirimidina en orina de 24 horas.

Purinas y pirimidinas	Intervalo de referencia, µmol/mmol creatinina	Paciente	Hermano
Uracilo	3,26–28,72	1,05	1,39
Timina	3,29–22,46	0,34	0,41
Hipoxantina	7,94–92,95	14,44	17,00
Xantina	0,02–103,35	108,35	117,41
Ácido úrico	615,34–5712,26	12,15	15,86
Uridina	29,21–181,00	0,76	0,73
Desoxiadenosina	2,84–33,74	10,89	17,10
Adenosina	2,46–11,30	0,29	0,25
Desoxiguanosina	0,35–5,07	0,05	0,05
Inosina	0,48–15,62	0,59	0,45
Guanosina	0,41–8,16	0,15	0,08
Desoxiuridina	1,45–31,21	0,25	0,52
Timidina	1,37–21,49	0,17	0,21

### Determinación de componentes en suero y orina

Se cuantificó el ácido úrico en suero y orina con el método de la uricasa (Advia 2400; Siemens Medical Solutions Diagnostics, Los Ángeles, CA, EE.UU), confirmándose con el método de la urato oxidasa (Dimension EXL; Siemens Medical Solutions Diagnostics, Los Ángeles, CA, EE.UU). Se determinaron las purinas y pirimidinas en papel de orina de 24 horas mediante espectrometría de masas (API 4000 Sciex Applied Biosystems) [11].

Se cuantificaron los niveles urinarios de N1-metil-2-piridona-5-carboxamida (2PY) y N1-metil-4-piridona-5-carboxamida (4PY) mediante HPLC en el Purine Research Laboratory (St Thomas’ Hospital NHS Foundation Trust, London, Reino Unido).

### Análisis genético – Secuenciación de alto rendimiento (NGS)

La NGS se realizó con el sistema SureSelectXT Custom para capturar las regiones codificantes y flanqueantes intrónicas de los genes *XDH* y *MOCOS*. Se reunieron las muestras y se marcaron con un código de barras. Se secuenciaron las genotecas capturadas en un secuenciador Ion Proton System empleando el sistema Ion PI™ Hi-Q™ y el Ion PI Chip (ThermoFisher Scientific). Las secuencias se alinearon con la secuencia del genoma humano (GRCh37/hg19) empleando el programa Torrent Mapping Alignment. Tras mapear la secuencia, se recogieron y anotaron las variantes con los siguientes programas: Torrent Variant Caller (TVC 5.2-25), GATK v3.7-0, Picard 2.9.0-1-SNAPSHOT, BEDtools v2.26.0, SAMtools 1.4, ExomeDepth 1.1.10.

## Resultados

### Determinación de componentes en suero y orina

Los niveles séricos de ácido úrico fueron inferiores a 5,95 μmol/L (intervalo de referencia: 142,75–356,88 μmol/L). La excreción urinaria de ácido úrico fue de 12,15 μmol/mmol creatinina (intervalo de referencia: 615,34–5712,26 μmol/mmol creatinina). La excreción urinaria de xantina e hipoxantina fue de 108,35 μmol/mmol creatinina (intervalo de referencia: 0,02–103,35 μmol/mmol creatinina) y 14,44 μmol/mmol creatinina (intervalo de referencia: 7,94-92,95 μmol/mmol creatinina), respectivamente.

Los niveles de 2PY determinados en una segunda muestra de orina fueron de 15,9 μmol/mmol creatinina, siendo imposible medir la 4PY posiblemente por una dilución de la muestra (1,7 mmol creatinina/L).

### Análisis genético – secuenciación de alto rendimiento

El paciente presentaba una mutación puntual en homocigosis de G a C en la posición menos 1 del intrón 23 del gen *XDH* (NM_000379.3(XDH):c2545-1G>C). Se trata de una nueva mutación puntual en el gen *XDH*, considerado potencialmente patogénico *in silico*. Esta mutación en un intrón podría influir en el proceso de corte y empalme, modificando la transcripción de ARN, dando lugar al déficit de *XDH* y al fenotipo de nuestro paciente. No identificamos ninguna otra mutación en este gen ni en *MOCOS*, por lo que se descartó la xantinuria tipo II.

## Discusión

La incidencia anual de la xantinuria hereditaria oscila entre 1:6000 y 1:69000, con más de 150 pacientes diagnosticados [12]. En España solo se han descrito algunos casos cuando, basándonos en los datos demográficos actuales, debería haber una incidencia superior a los 600 casos [13], [14], [15], [16]. Esto demuestra un infradiagnóstico de esta patología, que se suele manifestar y detectar en los análisis rutinarios a través de la hipouricemia.

El diagnóstico de HX se basa en niveles extremadamente bajos de ácido úrico en suero y orina, y una elevada excreción de xantina tras descartar otras causas. El diagnóstico diferencial de la hipouricemia se basa en la excreción de ácido úrico [17]. Son posibles causas de hipouricemia y de una elevada excreción de ácido úrico los tratamientos con salicilatos, la administración de medios de contraste intravenosos, la nutrición parental, la presencia de una neoplasia, la enfermedad de Wilson, SIADH, el síndrome de Fanconi, la cistinosis, el mieloma, la presencia de metales pesados, la hipouricemia renal hereditaria y diabetes mellitus. Todas ellas fueron descartadas por la baja excreción de ácido úrico [18, 19]. Por otro lado, se han descrito concentraciones disminuidas de ácido úrico en suero y orina en neoplasias, hepatopatías graves, tratamientos con inhibidores de la xantina oxidasa y xantinuria. El paciente permaneció asintomático, con valores normales de enzimas hepáticas, sin haber recibido tratamiento con hipouricemiantes. Esta información orientó el diagnóstico hacia un nuevo caso de xantinuria.

En segundo lugar, el tipo de HX se determina mediante la prueba de sobrecarga de alopurinol [20]. En voluntarios sanos, el oxipurinol es excretado en orina tras la administración de alopurinol, ya que este es metabolizado tanto por XDH/XO como por AOX. Por el contrario, en pacientes con xantinuria tipo II no se detecta oxipurinol ni en suero ni en orina, ya que no hay actividad en ninguna de ellas. Por otro lado, en pacientes con xantinuria tipo I, se puede detectar oxipurinol, ya que aún existe actividad en AOX, pero no en XDH/XO. Finalmente, la presencia de HX se confirma determinando la actividad de XDH/XO en la mucosa duodenal. Niveles muy bajos de actividad de XDH confirmarían el diagnóstico de xantinuria. Esta opción se descartó por la invasividad del procedimiento.

Recientemente, se ha descrito un algoritmo diagnóstico no invasivo basado en tres pasos [10]. El primer paso se basa en la detección de niveles bajos de ácido úrico en suero y orina, y una elevada concentración de xantina en orina. El segundo paso consiste en la tipificación de HX por medio de la metabolómica urinaria. Según la literatura, AOX participa en el catabolismo de la N1-metilnicotinamida, y los metabolitos finales N1-metil-2-piridona-5-carboxamida (2PY) y N1-metil-4-piridona-5-carboxamida (4PY) podrían emplearse como biomarcadores para distinguir la HX tipo I del tipo II. En la HX tipo II se observan niveles bajos de 2PY y 4PY, ya que no existe actividad de AOX [21]. El último paso es la confirmación de una mutación en el gen *XDH/XO* o en *MOCOS*.

En nuestro caso, la hipouricemia y la baja excreción de ácido úrico se confirmaron con dos métodos junto con la excreción de 2PY, lo que refuerza el diagnóstico de HX tipo I, que se confirmó con el hallazgo de la mutación en homocigosis anteriormente descrita.

Así mismo, la hipouricemia detectada en dos de sus hermanos apoya el componente hereditario de esta patología, aunque ambos rehusaron someterse a las pruebas genéticas. De este modo, se pudo realizar un diagnóstico temprano en el paciente, previniendo complicaciones renales derivadas de los cálculos renales ya presentes, así como permitiendo proporcionar al paciente y a sus familiares recomendaciones dietéticas y seguimiento adecuado.

## Conclusiones

Describimos un caso de xantinuria tipo I derivado de la mutación en homocigosis NM_000379.3(XDH):c2545-1G>C. Se trata de una nueva mutación puntual al parecer responsable del fenotipo del paciente, aunque es necesario investigar más casos para confirmar dicha asociación.
